# INTERSECTIONAL AGEISM: HOW BLACK OLDER ADULTS ENVISION A FUTURE WITH CONVERSATIONAL TECHNOLOGIES

**DOI:** 10.1093/geroni/igae098.1439

**Published:** 2024-12-31

**Authors:** Robin Brewer, Christina Harrington, Courtney Heldreth

**Affiliations:** University of Michigan, Ann Arbor, Michigan, United States; Google, Atlanta, Georgia, United States; Google, Seattle, Washington, United States

## Abstract

Dominant narratives of aging often center whiteness. However, older age can intersect with other identities that experience structural inequities and exacerbate ageism. In this study, we explore what it means to design technologies that are fair for historically minoritized older adults. We conducted design workshops with 16 Black older adults, asking how they envision older age and race represented in one form of AI system - conversational technologies. We highlight concerns around sharing age-related data, potential ageist outcomes that intersect with racism, and desires for authentically representing older age and race. We discuss these findings with a lens on how ageism discourse can exclude certain groups and how we might mitigate age-related AI harms in the technology design process by incorporating other values such as authenticity.

